# Spiking Neuron with Sensing Coil Based on a Volatile Memristor

**DOI:** 10.3390/s26072144

**Published:** 2026-03-31

**Authors:** Timur Karimov, Vyacheslav Rybin, Vasiliy Pchelko, Alexander Mikhailov, Yulia Bobrova, Denis Butusov

**Affiliations:** 1 Youth Research Institute, Saint Petersburg Electrotechnical University “LETI”, Professora Popova St. 5F, Saint Petersburg 197022, Russia; tikarimov@etu.ru; 2 Department of Computer-Aided Design, Saint Petersburg Electrotechnical University “LETI”, Professora Popova St. 5F, Saint Petersburg 197022, Russia; vgrybin@etu.ru (V.R.); vapchyolko@stud.etu.ru (V.P.); aamikhailov@etu.ru (A.M.); jobobrova@etu.ru (Y.B.)

**Keywords:** volatile memristor, spiking neural networks, sensory neuron, electromagnetic sensing, metal detection, proximity sensing, neuromorphic hardware

## Abstract

The convergence of sensing and processing is a critical frontier in the development of energy-efficient spiking edge intelligence. This paper presents a novel hardware implementation of a sensory neuron evolving from the leaky integrate-and-fire (LIF) model by coupling a volatile memristor with an LC tank circuit. The proposed memristor–resistor–inductor–capacitor (MRLC) neuron embeds electromagnetic sensing directly into neuronal dynamics, enabling direct transduction of proximity information into spike trains. We demonstrate that the circuit functions as a metal-sensitive proximity sensor with spiking output in both simulation and physical experiments. Moreover, the MRLC neuron exhibits rich dynamical regimes, including regular spiking, bursting with 2–5 spikes per burst, and quasi-chaotic behavior, as well as sensing memory provided by hysteresis-like multistability, which is a notable advancement over simple rate-encoding LIF neurons.

## 1. Introduction

Spiking neural networks (SNNs) represent the third generation of neural networks, most closely resembling biological neural systems [1]. They are expected to supplement or even displace the currently widespread second-generation networks based on static neuron activation functions. Unlike von Neumann architectures constrained by the processor-memory data transfer bottleneck, neuromorphic hardware enables highly distributed in-memory processing, leading to substantial reductions in latency and power consumption [2]. An important step toward neuromorphic artificial intelligence (AI) is the implementation of sensory neurons that directly transduce measured quantities into spike trains, i.e., into a form processable by SNNs [3]. Various algorithms may be used to convert the signal from conventional sensors into a format suitable for spiking neural networks; this is implemented, for example, in odor recognition systems based on standard gas detectors [4,5]. However, such encoding often entails information loss [6]. In contrast, neuromorphic sensors used in conjunction with spiking neural networks not only overcome the aforementioned encoding problem but also offer advantages in speed, efficiency, and fault tolerance over devices implemented with traditional technologies [7].

State-of-the-art neuromorphic sensors prioritize energy efficiency and circuit simplicity; therefore, they most often implement simple dynamic neurons, such as the leaky integrate-and-fire (LIF) model. Recent examples of such sensors include an aqueous ammonia detector [8], a force sensor based on a force-sensitive resistor [9], and an artificial nose element consisting of a chemoresistive gas sensor and a single CMOS transistor [10]. A special interest goes to sensors based on promising energy-efficient devices—volatile memristors—capable of emulating ion channels in biological neurons and thus providing neuromorphic dynamics with very simple circuitry [11,12,13]. Examples of such developments include sensors for chemical [14] and thermal [15] analysis, a multifunctional neuromorphic sensor with resistive transduction [16], and others. Beside sensors, memristors find applications in spiking neural networks and in-memory computing for processing results of neuromorphic measurements. A comprehensive review of memristor applications confirms the growing interest in these devices for neuromorphic hardware implementations [17,18,19].

However, efforts in neuromorphic sensing have mainly focused on visual and tactile modalities, while the design of spiking neurons with electromagnetic field (EMF) perception remains largely unexplored. An example of a spiking sensor based on Josephson junctions capable of directly sensing magnetic flux was recently proposed in [20]. Compared to this design, memristor-based spiking circuits offer an even more promising pathway to address EMF sensing, as they can operate at normal temperatures, demonstrate a wide-range response, and have compact size and low cost.

In this work, we present a spiking neuron with a sensing coil based on a volatile memristor, coupled to an LC via bottom electrode. This configuration allows electromagnetic perturbations to directly modulate the memristor switching. As a result, the neuron generates spike trains in response to the external electromagnetic field. The proposed circuit can be used for metal detection, proximity sensing, EMF strength measurement, and other tasks.

The key contribution and novelty of this work are as follows:

1. We evolve the design of a sensory LIF neuron with a volatile memristor by incorporating an LC tank into the circuit, thereby enabling direct electromagnetic field perception by the neuron.
  We demonstrate the variety of dynamical modes of the proposed neuron, including regular spiking, chaotic spiking, and bursting modes.
2. To perform hardware prototyping of the proposed circuit, we design and implement a volatile memristor emulator with a conductive channel produced by CMOS analog key.
3. By tuning the circuit parameters for a specific volatile memristor, namely, AND-TS [21], and an industrial 360 µH proximity sensing coil, we show in both simulation and experiment that changes in the distance between the sensing coil and a metal object produce recognizable spiking patterns.

The rest of this paper is organized as follows. Section 2 presents the device physics and circuit model. Section 3 provides simulation and experimental results. Section 4 discusses counterparts, applications, and limitations, and Section 5 concludes the paper.

## 2. Materials and Methods

**Figure 1:**
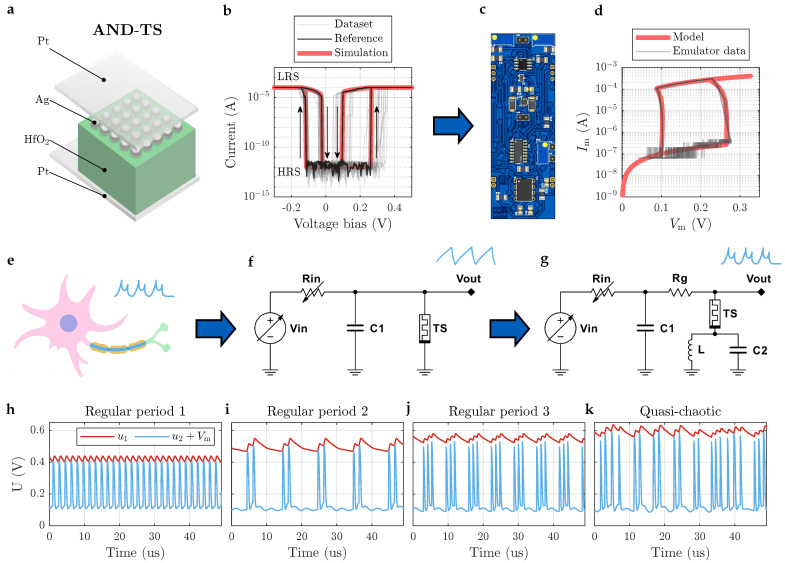
Framework of the study. (**a**) An example of a volatile memristor: AND-TS device (ordered Ag nanodots based threshold switch). It consists of Pt bottom electrode, 5 nm HfO_2_ dielectric layer, spherical polycrystalline Ag nanodots layer, and Pt top electrode. (**b**) Bidirectional threshold switching behavior of AND-TS under voltage sweep. (**c**) Volatile memristor emulator board from off-the-shelf components designed and adjusted to precisely reproduce AND-TS dynamical behavior, as is shown (**d**) in IV hysteretic curves comparison. (**e**) Biological sensory neurons often show a bursting behavior [22], (**f**) while theshold switching (TS) memristor-based LIF neurons are capable of producing only simple regular spikes. By supplementing the schematic with sensitive LC tank, (**g**) neuron obtains an ability not only to sense electromagnetic field, but also exhibit complex biomorphic behavior. (**h**) An example of regular spiking, (**i**) period–2, (**j**) period–3 and (**k**) quasi–chaotic spiking found in enhanced neuron model (**g**).

Figure 1 illustrates the framework used in this study. It is clarified in the following subsections.

### 2.1. Sensory LIF Neuron for Multimodal Sensing

Several recent works present the memristive leaky integrate-and-fire (LIF) sensory neurons [16,23,24], which serve as a starting point for developing more sophisticated designs. The LIF neuron circuit comprises a topology with a single capacitive node driven by a voltage source through a series load resistor, where a volatile memristor is connected in parallel to the capacitor. In [16], epitaxial VO_2_ memristor is used, while [23,24] rely on NbO_x_ devices; generally, any threshold switching memristor is suitable.

The schematic of this circuit is presented in Figure 1f. When external voltage $V_{in}$ is applied to it, the capacitor charges through the load resistor $R_{in}$. Once the voltage across the memristor exceeds the threshold voltage (e.g., $V_{\mathrm{th}}\approx 1.35$ V for VO_2_ device [16]), the memristor switches from a high resistance state (HRS) to a low resistance state (LRS). This triggers a “fire” event, generating an electrical spike as the capacitor rapidly discharges through the low-resistance channel. When the voltage drops below the holding voltage ($V_{\mathrm{hold}}\approx 0.85$ V for VO_2_ device), the device resets to HRS, and the charging cycle resumes. The spiking frequency *f* is determined by the charging and discharging time constants, which are functions of the series resistance, input voltage, parallel capacitance, and intrinsic timing constant of the memristor. Specifically, the frequency decreases with increasing load resistance or capacitance, and increases with higher input voltage, allowing for tunable neuronal dynamics. The cycle-to-cycle uniformity of TS device provides the accuracy of the sensor.

The dynamics of the memristive LIF neuron are described by the second-order ODE. Let $u_{1}$ denote the membrane potential across the capacitor $C_{1}$, and *x* denote the internal state variable of the memristor. As it is connected directly to the capacitor and both are connected to ground, the voltage across the memristor is $V_{m}=u_{1}$. Application of Kirchhoff’s current law at the capacitor node gives:

(1) $$C_{1}\frac{du_{1}}{dt}=\frac{V_{\mathrm{in}}-u_{1}}{R_{\mathrm{in}}}-I_{m}\left(u_{1},x\right).$$

Memristor current $I_{m}$ and the evolution of the internal state variable *x* may be expressed by magnetic flux-controlled memristor (MFCM) formalism:

(2) $$\left\{\begin{array}{cc} I_{m} & =g(V_{m},x),\\ \frac{dx}{dt} & =f(V_{m},x), \end{array}\right.$$

The specific forms of g(·) and f(·) depend on the physical characteristics of the volatile threshold switching memristor and its particular mathematical model. Although MFCM assumes x=ϕ (magnetic flux), physically, the switching mechanism of real memristors is based on the reversible breakdown of a thin-film layered structure.

To function as a multimodal sensor, the circuit is configured so that the fixed load resistor $R_{in}$ is replaced by calibrating resistor $R_{c}$ connected in series with external sensory elements whose resistance varies in response to physical stimuli. This design allows direct encoding of physical signals into spike rate with frequency *f*. Compatibility with various sensor modalities was demonstrated [16,24]:

- Tactile perception: a graphene aerogel pressure sensor decreases its resistance in response to the applied pressure, which results in monotonically increasing spiking frequency.
- Vision perception: a light sensor, such as GL3537-1 photoresistor, reacts to the illuminance increase by decreasing its resistance, resulting in higher spike rates.
- Temperature perception: NTC temperature sensor lowers resistance as a response to rising temperature, which increases firing frequency.
- Curvature perception: a curvature sensor such as FLEX4.5 modulates resistance based on bending.
- Chemical perception: the connection of a-IGZO thin-film transistor at the input stage of a circuit allows an inhibitory property under NO_2_ gas stimulation.

The disadvantages of the described LIF neuron include the very simple dynamics (the possible coding types include only spike rate and time-to-first spike). This circuit is also unsuitable for electromagnetic measurements, as it does not include an inductive element. The modification proposed below addresses these issues.

### 2.2. Sensory MRLC Neuron

The proposed neuron circuit is presented in Figure 1g. According to the used components, we will refer to it as the memristor–resistor–inductor–capacitor (MRLC) neuron. The core of the neuron dynamics relies on the interaction between two capacitive nodes, an inductive branch, and a volatile threshold switching memristor. The system is described by a set of five coupled ordinary differential equations (ODEs): three for the circuit, and two for the second-order memristor model.

Let $u_{1}$ and $u_{2}$ denote the voltages across capacitors $C_{1}$ and $C_{2}$, respectively, and $i_{L}$ denote the current flowing through the inductor *L*. The input voltage is defined as $V_{in}$, supplied through a series resistor $R_{in}$. The memristor is connected in series with a resistor $R_{g}$ and grounded by a capacitor $C_{2}$ and an inductor *L*.

Applying Kirchhoff’s laws, the time evolution of the circuit state variables is governed by

(3) $$\left\{\begin{array}{cc} C_{1}\frac{du_{1}}{dt} & =\frac{V_{in}-u_{1}}{R_{in}}-\frac{u_{1}-u_{2}-V_{m}}{R_{g}},\\ C_{2}\frac{du_{2}}{dt} & =-i_{L}+\frac{u_{1}-u_{2}-V_{m}}{R_{g}},\\ L\frac{di_{L}}{dt} & =u_{2}. \end{array}\right.$$

Here, the term $\left(u_{1}-u_{2}-V_{m}\right)/R_{G}$ represents the current flowing through the memristor branch, calculated via the voltage drop across the resistor $R_{G}$.

The voltage drop across the memristor, denoted as $V_{m}$, is determined algebraically by the voltage divider formed by the memristor resistance $R_{mem}$ and the series resistor $R_{g}$:

(4) $$V_{m}=\frac{R_{m}}{R_{g}+R_{m}}\left(u_{1}-u_{2}\right).$$

The memristor behavior is characterized by two internal dynamic variables: the state variable x∈[0,1] and the memristance $R_{mem}$. Unlike non-volatile models where resistance is an algebraic function of the state, this volatile AND-TS model employs differential equations for both variables, allowing for relaxation dynamics.

The state variable *x* evolves based on the instantaneous memristor voltage $V_{m}$ relative to threshold voltages $V_{on}$ and $V_{off}$. The dynamics are defined by the function $F_{MEM}$:

(5) $$\frac{dx}{dt}=\left\{\begin{array}{cc} \tau_{x}\left(1-x\right), & \mathrm{if}\;V_{m}\geq V_{on}\;\left(\mathrm{set}\right)\\ -\tau_{x}x, & \mathrm{if}\;V_{m}\leq V_{off}\;\left(\mathrm{reset}\right)\\ 0, & \mathrm{otherwise}\;(\mathrm{hold}) \end{array}\right.$$

where $k_{x}$ is the state switching rate constant.

The memristance $R_{mem}$ relaxes towards either the LRS ($R_{on}$) and HRS ($R_{off}$) depending on the value of the state variable *x*. This is described by the function $R_{MEM}$:

(6) $$\frac{dR_{mem}}{dt}=\left\{\begin{array}{cc} \tau_{R}\left(R_{on}-R_{mem}\right), & \mathrm{if}\;x>0.5\\ \tau_{R}\left(R_{off}-R_{mem}\right), & \mathrm{if}\;x\leq 0.5 \end{array}\right.$$

where $\tau_{R}$ is the resistance settling time constant.

Formulas (5) and (6) follow the standard phenomenological approach for volatile threshold switching memristors [11,25], where the state variable dynamics are governed by threshold-dependent relaxation processes. Similar differential equation-based models have been successfully applied to NbO_x_ and VO_2_ threshold devices [12]. The only need for a new simplified formulation is a strict requirement for numerical stability of the model, which is not satisfied with the advanced AND-TS model [13]. With that, a comparison between the advanced and simplified model shows that the new model keeps realistic non-immediate resistance switching and slightly differs only in transients.

The specific physical parameters used in the model are listed in Table 1. For the experiments, we used the AND-TS (Ag nanodots-based threshold switch) device which features a metal–insulator–metal stack with highly ordered Ag nanodots as the active electrode [21]. AND-TS allows stable volatile threshold switching with bidirectional operation in a compact 5 × 5 µm^2^ footprint, suitable for a number of applications, from volatile memory to quasi-Hodgkin–Huxley dynamical neuron design [13].

The system dynamics, including the phase space trajectories of $\left(u_{2},u_{1}\right)$ and $\left(u_{2}+V_{mem},u_{1}\right)$, are illustrated in Figure 1g–k. The volatile nature of the memristor introduces complex spiking and bursting behaviors characteristic of neuromorphic hardware implementations.

The inductance model for the sensing coil as the function from distance *d*, based on measurements between the coil and the steel plate, is given by

(7) $$L\left(d\right)=\frac{1.59\cdot 10^{-11}}{{\left(d+6.996\cdot 10^{-3}\right)}^{3}}+3.585\cdot 10^{-4},$$

where *d* is the distance in m, and L(d) is inductance in H. The inductance–distance relationship L(d) given by (7) is an empirical curve fit derived from measurements with a specific 360 µH industrial coil and a steel plate target. The model parameters, i.e., coefficients and exponent, depend on coil geometry, number of turns, core material, and target object properties such as permeability and conductivity. For different coil configurations or target materials, the model would require recalibration. This limits the generality of the quantitative results but allows fine correspondence between numerical simulation and experiment with the particular experimental setup.

### 2.3. Volatile Memristor Emulator Circuit

For prototyping circuits with volatile memristors, there is a problem in that such devices nowadays are mostly experimental and not available off the shelf. To face this challenge, we developed a simple emulator circuit with realistic behavior, which uses conventional components.

**Figure 2:**
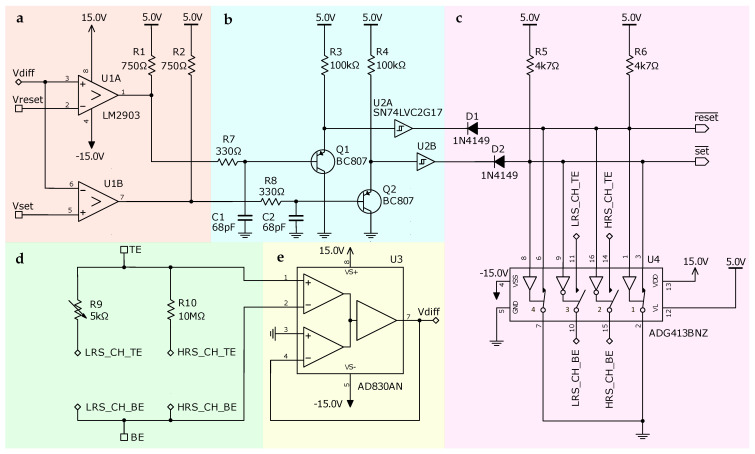
Schematic diagram of the hardware volatile memristor emulator. The circuit consists of the following stages: (**a**) comparator aimed to detect crossing of switching thresholds; (**b**) level-matching and debouncing stage (**c**); SR-latch based on analog key ADG413 which controls circuit conductance; (**d**) conductive channel emulator stage; (**e**) differential voltage detection stage based on wideband differencing amplifier AD830.

The circuit, presented in Figure 2, provides a versatile framework for emulation of several classes of memristive threshold selectors. Its operation is organized in five functional blocks. Block **a** is a threshold-comparison stage, employing paired LM2903 comparators ($U_{1A}$ and $U_{1B}$), that monitors the differential voltage $V_{\mathrm{diff}}$ against the set ($V_{\mathrm{set}}$) and reset ($V_{\mathrm{reset}}$) thresholds and implements inverted logic of operation required for proper switching of the trigger stage **c**. Featuring open-collector outputs, each comparator channel requires an external resistor, $R_{1}$ and $R_{2}$, pulled up to logical “1” level (5V). When $V_{\mathrm{diff}}<V_{\mathrm{reset}}$, the internal output transistor of $U_{1A}$ drives the set line to low power supply −15V; otherwise, the transistor pulls the line to the logic high level. Similarly, $U_{1B}$ shifts the line down when $V_{\mathrm{diff}}>V_{\mathrm{set}}$. Block **b** is the level matching and debouncing stage, ensuring the signal integrity between the comparator working from a ±15V power supply and the analog key with TTL-compatible logic input. Each signal line incorporates a conditioning circuit consisting of a PNP transistor BC807, an RC filter, and a Schmitt trigger implemented via two-channel gate SN74LVC2G17D ($U_{2}$). The PNP transistors $Q_{1}$ and $Q_{2}$ serve as level shifters, translating the comparator’s low level −15V to ground level, i.e., logic “0”. Following the transistor stage, RC low-pass filters implemented via $R_{3}$-$C_{1}$ and $R_{4}$-$C_{2}$ pairs, respectively, remove high-frequency interference, which may be produced by comparators in case of a noisy differential signal and result in false triggering. The resistor–capacitor time constant is selected in such a way to maintain acceptable time response for overall circuit operation. The Schmitt trigger gates with hysteresis convert the filtered analog signal into a clean logical waveform. The hysteresis characteristic provides additional noise immunity by establishing distinct thresholds for rising and falling edges.

Block **c** is a state-retention latch implemented via the analog CMOS key ADG413 using its channels 1 and 4, maintaining one of two stable states to control the conductive channel.

Block **d** is a conductive channel emulated by the ADG413 chip’s channels 2 and 3, with resistors $R_{9}$ and $R_{10}$ providing circuit conductance for a low-resistance state (LRS) and a high-resistance state (HRS), respectively, between the top electrode (TE) and the bottom electrode (BE). The use of an analog key for emulating a conductive path allows working in both positive and negative voltages applied to the emulator, in contrast to using MOSFETs.

Block **e** is a differential voltage detector between TE and BE contacts based on AD830 fully differential amplifier, which generates the signal $V_{\mathrm{diff}}$.

Overall circuit design uses components with bandwidth ≥1 MHz. In particular, Schmitt trigger has an average rise and fall time of 10 ns/V, DG413 has a typical turn-on and -off time of ≈100 ns, and AD830 has a bandwidth of 15 MHz. This ensures proper replication of real memristor dynamical properties; e.g., for AND-TS, set and reset delays are about 0.2 µs. To slow down the emulator circuit, capacitor values $C_{1}$ and $C_{2}$ may be adjusted. For the particular values, the settling time of 5V at the transistor $Q_{1}$ base is $\tau_{90\%}=\left(R_{1}+R_{7}\right)\cdot C_{1}\cdot \ln\left(10\right)\approx$ 0.2 µs.

The circuit board was prototyped using the toner transfer method, a cost-effective technique for rapid PCB fabrication. Component lead holes and layer interconnections were drilled using a CNC milling machine with automated toolpath control. This combined toner-transfer and CNC-drilling workflow enables quick iteration of two-layer designs with reasonable resolution and mechanical accuracy, making it well-suited for laboratory-scale PCB development.

## 3. Results

### 3.1. MRLC Neuron Model Investigation

The dynamical properties of the proposed MRLC neuron were investigated through extensive numerical simulations prior to hardware implementation. The system of coupled ordinary differential equations (ODEs) governing the circuit dynamics, comprising three equations for the electrical node voltages $u_{1}$, $u_{2}$, and current $i_{L}$ (3), and two equations for the memristor’s internal state variables *x* (5) and $R_{mem}$ (6), was solved using an explicit fourth-order Runge–Kutta (RK4) method with a fixed time step of h=2 ns to ensure numerical stability and accurate resolution of the fast switching transients. These numerical experiments established the foundation for subsequent hardware validation.

The simulation framework included GPU-based nonlinear dynamics analysis toolbox [26]. This approach enabled efficient exploration of the parameter space and comprehensive bifurcation analysis, revealing the rich repertoire of spiking behavior of the model. Namely, found regimes include regular spiking, bursting with 2–5 spikes in a burst, and quasi-chaotic oscillations; some of them are presented in Figure 1h–k.

A set of numerical experiments was performed with the inductance–distance relationship L(d) derived from experimental measurements (7), aiming to characterize MRLC circuit response to changes in inductance.

**Figure 3:**
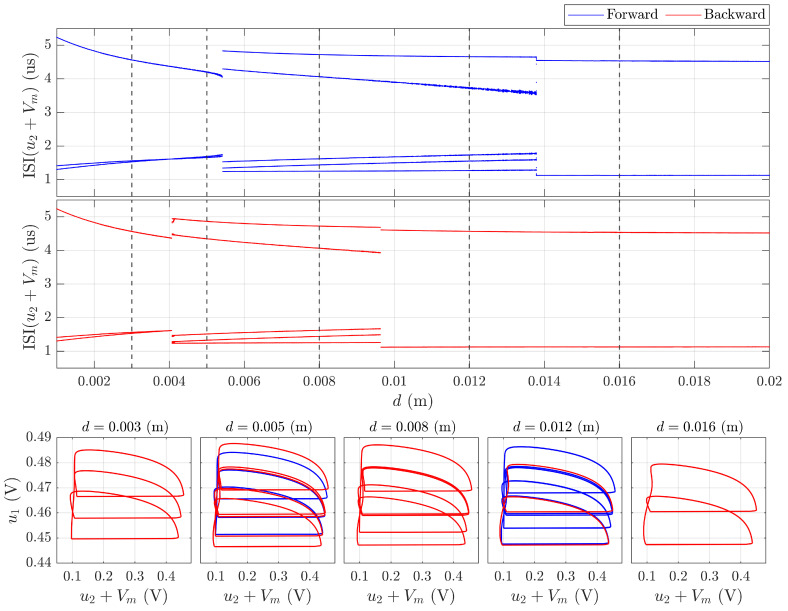
Periodic spiking analysis of the MRLC neuron dynamics as a response to distance *d* between sensing coil and steel plate. Interspike interval diagrams reveal bursting behavior with different numbers of spikes in bursts, seen as loops of attractors in the phase space. Hysteresis emerged from multistability between approaching (backward) and receding (forward) directions, enabling direction-sensitive proximity detection with intrinsic memory. Dashed lines correspond to phase portraits shown in lower panels.

Figure 3 presents a comprehensive analysis of these dynamics through interspike interval (ISI) diagrams and phase space attractors reconstructed from the membrane potential $u_{2}+V_{mem}$.

As the conductive target approaches the sensing coil (decreasing distance *d*), the system undergoes a sequence of bifurcations leading to distinct periodic regimes. At specific distance intervals and target moving history, the neuron exhibits stable period-2, period-3, period-4, and period-5 oscillations, each characterized by unique ISI distribution and attractors in the $\left(u_{1},u_{2}+V_{m}em\right)$ phase plane. The rapid transitions between bursts with different periods demonstrate the sensor’s ability to encode proximity information through complex temporal patterns beyond simple rate encoding.

A particularly notable phenomenon observed is the multistability of the sensor response when the target approaches the coil (referred to as the backward direction) versus when it recedes (forward direction). This directional dependence manifests as a hysteresis loop in the system’s dynamical behavior: for the same physical distance *d*, the neuron may exhibit different spiking patterns depending on the history of target movement. For instance, when approaching from a far distance, the system might maintain regular spiking until a critical distance where it abruptly transitions to period-3 oscillations; however, when receding from a close distance, the return to regular spiking occurs at a different threshold, preserving the periodic regime over a broader range. Examples of multistable attractors are presented for d=0.005m and d=0.012m. This useful hysteresis property is implemented in contactless limit switches by specialized circuitry, but in MRLC sensors it emerges directly from their nonlinear dynamics. From a practical perspective, the hysteretic behavior can be exploited for noise-immune detection, position sensing with directional discrimination, and implementation of simple decision-making directly at the sensor level without additional signal processing.

**Figure 4:**
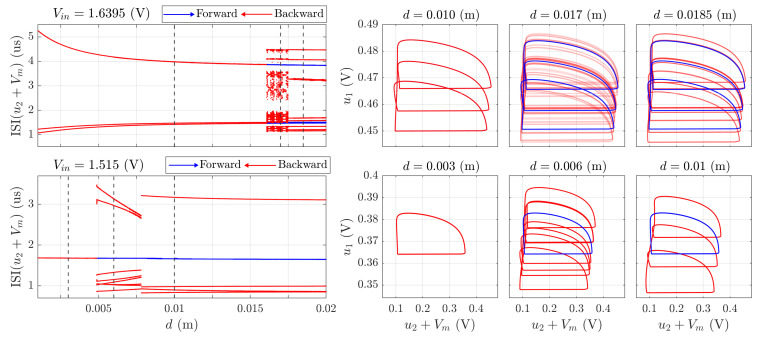
(**Upper Panel**): Quasi-chaotic spiking regimes in the MRLC neuron enable long-range proximity detection. Minor changes in inductance ΔL at large distances *d* produce measurable alterations in interspike interval distributions and phase space trajectories due to operation near bifurcation boundaries. (**Lower Panel**): “Dead mode” dynamics, i.e., the system’s collapse into a stable period-1 limit cycle as the conductive target approaches a critical distance. This regime represents minimal encoding complexity but maintains continuous oscillatory behavior, insignificantly reacting to the target. Dashed lines correspond to phase portraits shown in right panels.

Beyond the periodic bursting, the MRLC neuron exhibits complex dynamical regimes that can be classified as quasi-chaotic, which are characterized by apparently irregular spiking patterns that in fact correspond to oscillations with long periods; see Figure 4. These regimes emerge at specific parameter values where the system operates near bifurcation boundaries, resulting in hypersensitivity to minor parameter variations.

The quasi-chaotic dynamics prove particularly valuable for long-range proximity detection. When the conductive target is far from the sensing coil (large distances *d*), the absolute changes in inductance ΔL per unit displacement become exceedingly small, following the power-law decay described by (7), and are hardly distinguishable on the noisy background. However, the MRLC neuron operating in a quasi-chaotic regime exhibits sensitivity to these perturbations, i.e., minor changes in L(d) at the bifurcation boundary induce measurable shifts in the interspike interval distribution.

This sensitivity arises from the system’s operation near critical points where the Lyapunov exponents approach zero, creating an “edge of chaos” condition that amplifies small parameter variations into discernible dynamical changes. Consequently, the quasi-chaotic regime enables detection of conductive objects at greater distances than would be possible with simple rate-encoding LIF neurons.

**Figure 5:**
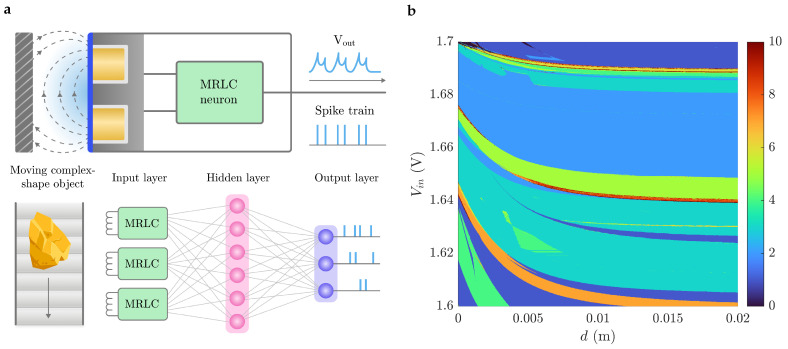
Conceptual overview of the MRLC neuron as an edge sensor for neuromorphic perception. (**a**) Neuron directly encodes proximity information into spike trains fed into a spiking neural network (SNN) for further processing, enabling end-to-end neuromorphic sensing-computing systems. (**b**) Dynamical regime map on the $V_{\mathrm{in}}$–*d* parameter plane, where colored regions indicate the number of distinct interspike intervals observed in the neuron’s output. Colors in the right panel correspond to the periodicity of signal.

Figure 5 presents the conceptual schematic of the MRLC neuron acting as edge sensor, feeding its output into spiking neural network, and a broad view on the dynamics on the $V_{in}$–*d* plane.

### 3.2. MRLC Neuron Experimental Investigation

#### 3.2.1. Setup

**Figure 6:**
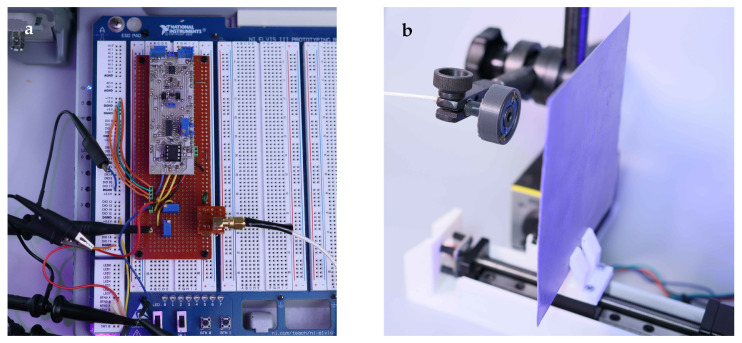
Photographs of the experimental setup: (**a**) a prototyping board with MRLC neuron circuit featured volatile memristor emulator; (**b**) industrial 360 µH proximity sensor coil and the target object—steel plate at the moving cart.

The experimental validation of the MRLC neuron was performed using a custom-built prototyping platform, as illustrated in Figure 6. The setup comprised two main components. First, a prototyping board of the MRLC neuron circuit, with the volatile memristor emulator described in Section 2.3. The board integrated the memristor emulator with the necessary passive components, i.e., capacitors $C_{1}$, $C_{2}$, coupling resistor $R_{g}$, and input resistor $R_{in}$, and was connected to the power supply (±15 V, 5 V) of the NI ELVIS III (National Instruments Corporation, Austin, TX, USA) station, arbitrary waveform generator Rigol DG1032Z (Rigol Technologies, Beijing, China) for feeding input voltage $V_{in}$ with 1 mV accuracy, a digital oscilloscope Rigol DS1054Z (Rigol Technologies, Beijing, China) for spike trains acquisition, and an analog oscilloscope S1-83 (PTO im. Lenina, Russia) for phase portrait observation with high resolution and zero latency. Second, the electromagnetic sensing front-end consisted of an industrial-grade proximity sensor coil with inductance *L* = 360 µH facing a steel plate mounted on a stepper-motor driven moving cart. The cart enabled controllable variation of the distance *d* between the coil and the steel plate with sub-millimeter accuracy, allowing systematic investigation of the sensor response across the full distance range $d\in [0,{ø}_{coil}]$. The complete assembly facilitated real-time observation of spike trains and phase portraits from the MRLC neuron output under varying parameters and proximity.

#### 3.2.2. Memristor Emulator Test

**Figure 7:**
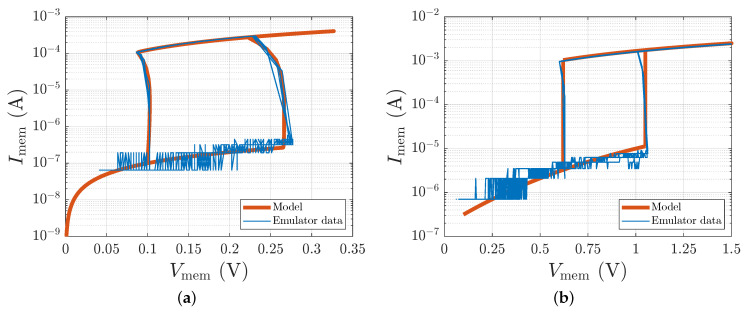
Memristor emulator board test ensures proper replication of different volatile memristors dynamics: (**a**) AND-TS device [21] and (**b**) sample of Mott memristor [12]. For the AND-TS device, the memristor parameters were set to $R_{\mathrm{on}}=800\;\Omega$, $R_{\mathrm{off}}=1.0\;M\Omega$, with threshold voltages $V_{\mathrm{on}}=0.266\;V$ and $V_{\mathrm{off}}=0.102\;V$. The Mott memristor emulation utilized $R_{\mathrm{on}}=600\;\Omega$, $R_{\mathrm{off}}=1.5\;M\Omega$, and switching thresholds $V_{\mathrm{on}}=1.052\;V$ and $V_{\mathrm{off}}=0.616\;V$. Numerical parameters were also used for simulation via (5) and (6).

To ensure the fidelity of the memristor emulator board, it was tested in emulation of two distinct classes of volatile threshold switching devices. The first class, metal-cation-based memristors, was presented by an Ag nanodots-based threshold switch (AND-TS) [21]. The second class comprised of memristors based on Mott materials, such as ${\mathrm{VO}}_{2}$, ${\mathrm{NbO}}_{x}$, ${\mathrm{NiO}}_{2}$, and ${\mathrm{TiO}}_{x}$, as insulating switching layers, was presented by the sample device from [12]. The emulator board was configured to replicate both the AND-TS device and a representative Mott memristor, with numerical parameters for the simulations following the state variable dynamics described by (5) and (6). As shown in Figure 7, the measured current-voltage characteristics of the emulator board closely match the reference device behaviors across multiple operational cycles. In particular, for the AND-TS device, the emulator accurately reproduces the bidirectional threshold switching with the set voltage $V_{\mathrm{on}}=0.267\;V$ and reset voltage $V_{\mathrm{off}}=0.1\;V$.

#### 3.2.3. Experimental Validation of MRLC Neuron Dynamics

**Figure 8:**
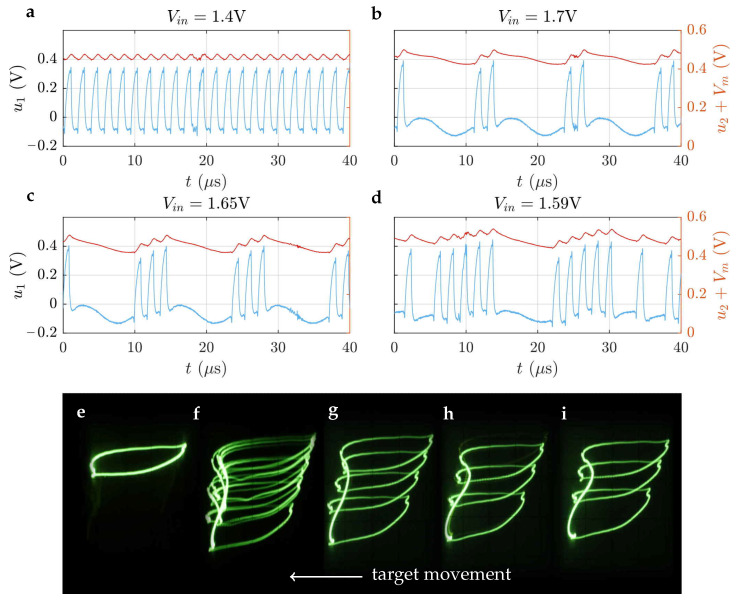
MRLC neuron experimental investigation. Upper plots show bursting modes obtained for different input voltages, (**a**) regular spiking mode, (**b**) bursting period-2 mode, (**c**) bursting period-3 mode, (**d**) and quasi-chaotic mode, which corresponds to simulation results in Figure 1h–k. Phase portraits captured from a cathode ray oscilloscope verify “dead mode” (**e**) achievement forecasted by the simulation in Figure 4. Period 3 bursting (**i**) at d=20 mm changes to period 4 (**g**) through flickering intermediate 3–4 mode (**h**). Then quasi-chaotic mode emerges (**f**), and at d=5 mm, dynamics collapses into period-1 cycle.

The experimental verification of the MRLC neuron was conducted by comparing the dynamical behavior of the physical circuit with numerical simulations across varying input voltages and sensing distances. As illustrated in Figure 8, the recordings from the digital oscilloscope demonstrate bursting modes obtained for different input voltages, close to the simulation results previously presented in Figure 1h–k.

Phase portraits captured from the cathode ray oscilloscope provided the visualization of the system’s dynamics in a phase space $u_{1}$–$u_{2}+V_{m}$. The presented photograph sequence demonstrates the achievement of the “dead mode” forecasted by simulation in Figure 4. It was obtained by varying the proximity of the steel target to the sensing coil. As the distance decreased from d=20 mm to d=5 mm, a characteristic transition pathway was observed: period-3 bursting (**i**) gradually evolved into period-4 (**g**), then to quasichaotic dynamics (**f**), and finally collapsed into a stable period-1 cycle. This experimentally observed sequence confirms the sensitivity of the MRLC neuron circuit to changes in inductance and validates the numerical predictions of multistability and hysteresis in the sensor response. Notably, the transition pathway observed in the hardware (period-3 → period-4 → quasi-chaotic → period-1) closely reflects the bifurcation structure obtained in numerical simulations; see Figure 4 (lower panel). Furthermore, the interspike intervals (ISI) measured in the experiment fall within the 1–10 µs range predicted by the model, demonstrating quantitative consistency between the proposed mathematical framework and the physical prototype. Minor deviations in threshold voltages were observed due to component tolerances in the emulator circuit (approximately 10% variation), but the topological features of the attractors and the sequence of dynamical regimes remained consistent across both domains.

## 4. Discussion

### 4.1. Comparison with Recent Counterparts

Table 2 demonstrates a comparison of the proposed sensor with designs presented in the literature. One may see that most of the existing neuromorphic sensors rely on LIF architecture with external sensitive transducers, while MRLC embeds sensing into neuronal dynamics through inductive coupling. Also, the MRLC design intends to operate at room temperature, unlike Josephson junctions-based sensor requiring cryogenic conditions [20], which is the only one neuromorphic sensor for electromagnetic field perception to date, as far as authors know.

### 4.2. Bursting Dynamics Mechanism

The complex bursting behavior of the MRLC neuron emerges primarily from the interaction between two distinct timescales: (1) $LC_{2}$ tank gated by memristor, and (2) the $R_{g}C_{1}$ network. This mechanism resembles bursting in the Hindmarsh–Rose neuron, arising from the interaction between fast membrane potential recovery (in MRLC neuron, the equivalent is the rapid charge–discharge cycle of the capacitor $C_{2}$ driven by the memristor switching), and slow adaptation current (equivalent to slower charge–discharge of $C_{1}$). Accordingly, an increase in $C_{1}$ value results in longer burst trains. Memristor relaxation dynamics ($\tau_{x}$, $\tau_{R}$) is also crucial for the bursting mechanism, but there is no simple dependence: bursts occur when switching is rather fast, not slow and not immediate. Also, timescale interaction together with nonlinear behavior of a memristor forms route to chaos in the model; in Appendix A, we give a brief overview of chaotic regimes. The exploration of the full palette of MRLC neuron behavior, as well as deep investigation of underlying dynamical mechanisms, are subjects for future research.

### 4.3. Possible Applications

The spiking output of the proposed MRLC sensory neuron is naturally compatible with spiking neural networks (SNNs). This eliminates the information loss associated with encoding signals from conventional sensors into spike trains, offering advantages in speed, efficiency, and fault tolerance for neuromorphic sensing systems [7].

Following the line of sensory LIF neurons use, MRLC neuron enables several key applications in the realm of edge intelligence:

- Metal detection and proximity sensing. The circuit functions as a standalone metal detector or proximity sensor. The experiments with an industrial 360 µH proximity sensing coil demonstrated the potential for detecting conductive objects through variations in the spiking output.
- Electromagnetic field measurement. The proposed design embeds electromagnetic sensing directly into neuronal dynamics, allowing for EMF strength measurement where external electromagnetic perturbations modulate the memristor switching.
- Low-power sensing. Utilizing volatile memristors (specifically the AND-TS device) allows for circuit simplicity and energy efficiency, operating at normal temperatures with a compact footprint suitable for embedded systems.

An important feature of a sensor is its response time. The MRLC neuron generates spikes with interspike intervals of 1–5 µs and encodes sensed information in complex spiking patterns rather than a single interspike interval. Thus, the effective response time to reliably detect a change in a measured quantity would depend on the time needed to observe a statistically significant change in interspike intervals or bursts’ structure. This time may be estimated as tens of microseconds, which is a relatively fast response in practice. For example, in the case of a proximity measurement task, an estimate of 100 µs is sufficient for observing most mechanical processes in industry and even vibration.

### 4.4. Limitations of the Study

While the proposed MRLC neuromorphic sensor demonstrates promising capabilities for electromagnetic sensing and, in particular, metal detection, several limitations should be acknowledged, as listed below.

Regime stability issues were not fully addressed. We did not take into account stochastic properties of memristor threshold voltages [21]. Also, thermal drift and other disturbances may be considerable factors in industrial environments, violating the desired dynamical behavior. For instance, the highly sensitive mode from Figure 4 was not stable due to interferences primarily from trimmer resistors and plug contacts, but other factors could also be of importance.

Complexity of spike pattern decoding: the neuron exhibits rich dynamical modes, including regular spiking, bursting, and quasi-chaotic behavior (Figure 1h–k). While this enhances sensing capabilities, it requires spiking neural network (SNN) decoding, in contrast to simple frequency-based encoding of LIF neuron models. Real-time classification of complex patterns may be challenging for processing units with limited performance.

Narrow sensing scenarios verified: the inductance model L(d) and experimental validation were derived using a steel plate as the target object. The sensor’s response to non-ferrous metals or external magnetic fields has not been characterized, which may limit generalizability across different industrial sensing scenarios but also provides space for further research.

Despite the mentioned limitations, the proposed MRLC neuron architecture represents a significant step toward compact and energy-efficient neuromorphic sensors for electromagnetic perception. The successful demonstration of spike-based proximity detection validates the feasibility of embedding previously unexplored sensing modalities directly into neuronal dynamics. The output of spiking sensors may be naturally connected to spiking neural networks, which have been shown to be capable of processing biological neural signals [27].

## 5. Conclusions

In this study, we present a novel sensory neuron that directly embeds electromagnetic sensing capabilities into spiking dynamics, and call it memristor–resistor–inductor–capacitor (MRLC) model. It enhances the classical leaky integrate-and-fire (LIF) model by coupling a volatile memristor with an LC tank circuit. The main goal was to overcome the limitations of existing memristive LIF sensors, particularly their unsuitability for electromagnetic measurement.

The work was performed in several distinct stages. First, we developed the mathematical framework for the MRLC neuron, deriving a system of five coupled ordinary differential equations to evaluate its complex dynamics. For this task, a novel model of an AND-TS volatile memristor with enhanced computational stability was proposed. Second, to bypass the lack of commercially available volatile memristors for the circuit validation, a hardware emulator was designed and prototyped using off-the-shelf components, which accurately reproduces the threshold switching behavior of the target device. Finally, the circuit’s functionality was validated through both numerical simulations and physical experiments, demonstrating its ability to perceive proximity between the sensing coil and the metal target.

An important feature of the proposed design is the richness of its dynamical regimes, including regular spiking, bursting with 2–5 spikes per burst, and complex quasi-chaotic behavior, which is a significant advancement over the simple spike-rate coding of standard LIF neurons. This brings the MRLC neuron closer to biological sensing neurons featuring bursting behavior. Quantitatively, the circuit leverages the fast switching of the AND-TS device and demonstates interspike intervals within the 1–10 μs range, ensuring a fast response suitable for various industrial applications.

Future work will focus on integrating the MRLC neuron with a spiking neural network (SNN) to demonstrate end-to-end neuromorphic perception and investigating the application of the sensor–SNN framework for complex sensing and measurements tasks.

## Figures and Tables

**Table 1:** MRLC neuron model parameters.

Symbol	Value	Description
$C_{1}$	1.0nF	Capacitance 1
$C_{2}$	1.395nF	Capacitance 2
*L*	360 µH	Inductance
$R_{in}$	10kΩ	Input resistance
$R_{G}$	2850Ω	Coupling resistance
$V_{\mathrm{in}}$	1.7V	Input voltage
$V_{\mathrm{on}}$	0.267V	Memristor set threshold
$V_{\mathrm{off}}$	0.1V	Memristor reset threshold
$R_{on}$	806Ω	Memristor low-resistance state
$R_{off}$	1MΩ	Memristor high-resistance state
$\tau_{x}$	$0.99\times 10^{6}\;s$	State variable time constant
$\tau_{R}$	$20\times 10^{6}\;s$	Resistance settling time constant

**Table 2:** Comparison of the proposed solution with other neuromorphic sensors.

Neuron Model	Measured Quantity	Core Device	Dynamical Features
LIF neuron for ammonia detection [8]	Chemical concentration (NH_3_)	CMOS transistor	Spike rate encoding
LIF neuron with force-sensitive resistor [9]	Pressure, force	NPN transistor	Spike rate encoding
Artificial olfactory LIF neuron [10]	Gas concentration (odor)	CMOS transistor	Spike rate encoding
LIF multifunctional sensory neuron [16]	Tactile, vision, temperature, curvature	Epitaxial VO_2_ memristor	Spike rate encoding
Thermal sensory LIF neuron [23]	Temperature	NbO_*x*_ volatile threshold device	Spike rate encoding
Neuron-SQUID (L-JJ model) [20]	Magnetic flux in quantum range	Superconductive Josephson junctions	Regimes including regular spiking, bursting mode, bursting plateau mode, etc.
MRLC neuron (this work)	Electromagnetic field, metal proximity	Ag nanodots threshold selector	Regular spiking, bursting, quasi-chaotic bursting, feasibility for spike rate encoding

**Figure A1:**
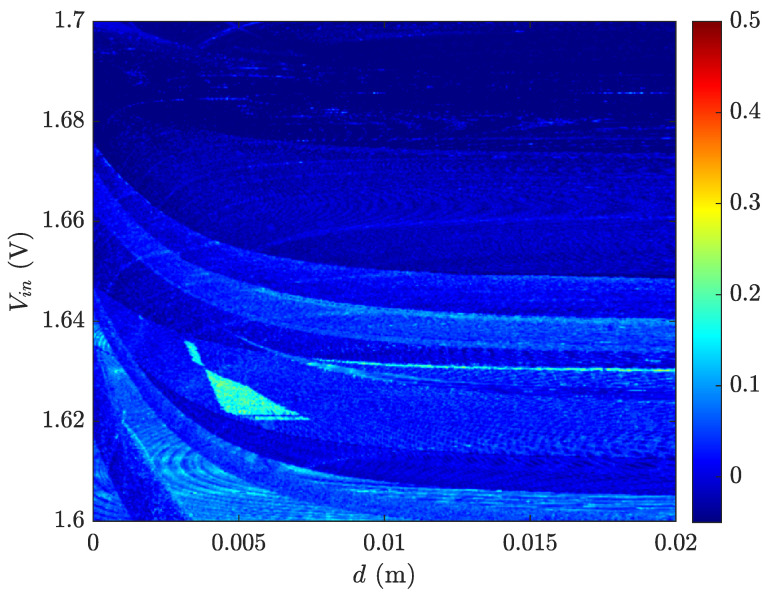
Largest Lyapunov exponent $\lambda_{1}$ distribution in the $V_{in}–d$ parameter plane built for µs time unit. Warmer-colored regions indicate higher $\lambda_{1}$ values, marking transitions to quasi-chaotic or chaotic dynamics; blue areas correspond to periodic oscillations.

## Data Availability

The original contributions presented in this study are included in the article. Further inquiries can be directed to the corresponding author.

## Abbreviations

The following abbreviations are used in this manuscript:

AND-TS	Argentum nanodot threshold selector
ANN	Artificial neural networks
CPU	Central processing unit
FPGA	Field-programmable gate array
GPU	Graphics processing unit
LSM	Liquid state machine
LSM-SOM	Liquid state machine stochastic organization mapping
MNIST	Modified National Institute of Standards and Technology
NPU	Neural processing unit
RC	Reservoir computing
SNN	Spiking neural networks
TPU	Tensor processing unit
TS-TD neuron	Threshold selector and tunnel diode based neuron

## Appendix A. Exploration of Chaotic Dynamics

To rigorously characterize the dynamical regimes, the largest Lyapunov exponent (LLE) was calculated to distinguish between chaotic and periodic behavior. Utilizing the computational framework described in [28], a two-parameter map of the Lyapunov exponents was constructed for the model using the same axes as in Figure 5b. The majority of the parameter space exhibits negative or near-zero LLE values $\lambda_{1}\leq 0$, corresponding to stable limit cycles (period-1 spiking) or periodic bursting regimes (period-2 to period-5). Distinct islands of positive but small LLE values $\lambda_{1}>0$ appear near bifurcation boundaries, where the system transitions between periodic modes. Partially, they correspond to the quasi-chaotic regimes described in Section 3.1, characterized by long-period, apparently irregular spike trains. The highest LLE values indicate weak chaos: trajectories separate gradually, preserving short-term predictability while enabling sensitivity to small parameter changes, which seems the most suitable case of chaos in measurement systems [29]. These quasi-chaos and weak chaos originate from complex interactions between four timescales (of $R_{g}C_{1}$ network, $LC_{2}$ tank, and the memristor’s switching $\tau_{x}$ and $\tau_{R}$) controlled by a nonlinear function, which is a known possible route [30].
